# Cascading Effects of Ocean Acidification in a Rocky Subtidal Community

**DOI:** 10.1371/journal.pone.0061978

**Published:** 2013-04-16

**Authors:** Valentina Asnaghi, Mariachiara Chiantore, Luisa Mangialajo, Frédéric Gazeau, Patrice Francour, Samir Alliouane, Jean-Pierre Gattuso

**Affiliations:** 1 DiSTAV - University of Genoa, Genoa, Italy; 2 Université de Nice-Sophia Antipolis, EA 4228 ECOMERS, Nice, France; 3 CNRS-INSU, Laboratoire d'Océanographie de Villefranche, Villefranche-sur-Mer, France; 4 Université Pierre et Marie Curie-Paris 6, Observatoire Océanologique de Villefranche, Villefranche-sur-Mer, France; University College Dublin, Ireland

## Abstract

Temperate marine rocky habitats may be alternatively characterized by well vegetated macroalgal assemblages or barren grounds, as a consequence of direct and indirect human impacts (*e.g*. overfishing) and grazing pressure by herbivorous organisms. In future scenarios of ocean acidification, calcifying organisms are expected to be less competitive: among these two key elements of the rocky subtidal food web, coralline algae and sea urchins. In order to highlight how the effects of increased *p*CO_2_ on individual calcifying species will be exacerbated by interactions with other trophic levels, we performed an experiment simultaneously testing ocean acidification effects on primary producers (calcifying and non-calcifying algae) and their grazers (sea urchins). Artificial communities, composed by juveniles of the sea urchin *Paracentrotus lividus* and calcifying (*Corallina elongata*) and non-calcifying (*Cystoseira amentacea* var *stricta*, *Dictyota dichotoma*) macroalgae, were subjected to *p*CO_2_ levels of 390, 550, 750 and 1000 µatm in the laboratory. Our study highlighted a direct *p*CO_2_ effect on coralline algae and on sea urchin defense from predation (test robustness). There was no direct effect on the non-calcifying macroalgae. More interestingly, we highlighted diet-mediated effects on test robustness and on the Aristotle's lantern size. In a future scenario of ocean acidification a decrease of sea urchins' density is expected, due to lower defense from predation, as a direct consequence of pH decrease, and to a reduced availability of calcifying macroalgae, important component of urchins' diet. The effects of ocean acidification may therefore be contrasting on well vegetated macroalgal assemblages and barren grounds: in the absence of other human impacts, a decrease of biodiversity can be predicted in vegetated macroalgal assemblages, whereas a lower density of sea urchin could help the recovery of shallow subtidal rocky areas affected by overfishing from barren grounds to assemblages dominated by fleshy macroalgae.

## Introduction

The partial pressure of CO_2_ (*p*CO_2_) in the atmosphere has increased by about 40% (267 to 384 ppm) since the beginning of the industrial revolution, leading to changes in the Earth's climate and in terrestrial ecosystems functioning [1]. This increase in atmospheric CO_2_, mainly due to anthropogenic emissions, led to a worldwide modification of the seawater carbonate system, causing a decrease in ocean pH. Mean surface ocean pH has decreased by approximately 0.1 unit between pre-industrial time and the 1990s [2]–[3], and a further decrease of approximately 0.4 units is predicted to occur by the end of the century [3]. This “ocean acidification” process may have profound impacts on marine biota, mostly through the direct effects of pH on inter-cellular transport mechanisms that control the physiology and metabolism of marine organisms [4] and the decreased availability of CO_3_^2−^, used by many species to build calcareous shells and skeletons. Temperate rocky reef communities, as coral reefs, are particularly threatened by ocean acidification, since most of their organisms use carbonates to build calcareous structures. These organisms are well known to be particularly sensitive to acidified conditions (*e.g.* [5]–[7]; see [8] for a recent review). The two main benthic calcifiers, corals and algae, secrete the most soluble forms of CaCO_3_. Corals secrete aragonite, while calcifying algae secrete either aragonite (green algae, such as *Halimeda* spp.) or high-magnesium calcite (coralline red algae) [9]). Experiments investigating the effects of elevated *p*CO_2_ on photosynthesis and/or calcification of calcifying algae show complex and species-specific responses, with variable results depending on the pathway of carbonate deposition and on its relative amount [9].

Along temperate Mediterranean coasts, shallow subtidal areas are dominated by macroalgae that provide a range of ecosystem services such as food source, shelter from predation and disturbance, proper settlement substrate and nursery for marine vertebrates and invertebrates. Macroalgal communities are particularly sensitive to anthropogenic disturbances and are, accordingly, used for ecological status assessment under the European Water Framework Directive 2000/60/EC [10]–[12]. Shifts between alternative states have already been reported [13]–[14], such as a loss of canopy-forming species (*i.e.* fucoids) in favour of turfs [15]–[16] or barrens of coralline algae [13], [17] in response to human pressures, *e.g.* urbanization of the coastline and overfishing (that favour proliferation of sea urchins).

Macroalgae also exhibit different sensitivities to increased sea temperature and ocean acidification [9], [18]–[20]. Non-calcifying algae generally show increased production and growth in response to elevated CO_2_ [21]–[23], while fucoid canopy algae, even though particularly sensitive to human impacts, are considered to be less sensitive to pH variations and their primary production might be even expected to increase in acidified waters [24]. Corallinales species are the dominant calcifying algal group and appear to be the most sensitive to ocean acidification: elevated *p*CO_2_ negatively affects their recruitment [25], growth [26] and calcification [7], [27]–[29]. They exhibit calcium carbonate dissolution and decreased surface percent cover at lower pH and may be less competitive for space, driving a shift from dominance of calcifying to non-calcifying algae [25], [30]–[32].

Sea urchins are generally the most effective benthic herbivores in shallow subtidal areas [33]. Although their grazing activity can facilitate the settlement of new species by providing patches of bare substrate, with an increase in biodiversity [34], when they are particularly abundant as a direct or indirect consequence of human disturbance (*e.g.* overfishing, [13]; date-mussel fishery, [17]), they can dramatically deplete non-calcifying algae, changing the seascape with the creation of extensive barren grounds dominated by coralline algae.

Sea urchins also show sensitivity to decreased pH: their carbonate structures (skeleton and grazing apparatus) are made up of the very soluble high-magnesium calcite, both in adult, juvenile and larval stages [35]–[37]. Adult sea urchin sensitivity to ocean acidification has been reported particularly by *in situ* records in naturally acidified areas [18], while laboratory experiments have mainly tested effects of low pH on larval stages or gene expression in the developmental process [36], [38]–[41].

Macroalgae and sea urchins therefore strongly interact to shape the ecological state of rocky ecosystems. Studies on their response to ocean acidification have been so far performed separately through laboratory experiments on isolated species [19], [21]–[22], [35], [42] and through *in situ* observations in naturally acidified areas [18], [30], [43], but it is crucial to assess the cascading effects of their individual responses [44].

The effects of elevate *p*CO_2_ on species interactions are still poorly known and the extent of consequences at the ecosystem level hard to assess. Most of the laboratory studies on ocean acidification have been focused on fitness and physiological processes of individual species [45]. Only a few studies, so far, have dealt with species interactions in naturally acidified locations [18], [43]–[44], that mimic future acidified scenarios but whose ecology is potentially affected by surrounding areas (see [44] for in depth discussion). Individual species fitness can affect communities and/or ecosystem functioning and biodiversity by cascade processes [8], [46] and *p*CO_2_ effects on upper trophic levels may be modulated (ameliorated or exacerbated) by indirect effects intrinsic to interactions with other trophic levels [47]. These effects cannot be ascertained without simultaneously testing response to ocean acidification on interacting species.

In this context, we performed one of the first, to our knowledge, laboratory experiments on ocean acidification taking into account species interactions. We used calcifying (*Corallina elongata*), and non-calcifying (the fucoid *Cystoseira amentacea* var. *stricta*, hereafter *Cystoseira amentacea*, and *Dictyota dichotoma*) macroalgae, and sea urchins (*Paracentrotus lividus*), under 4 different *p*CO_2_ conditions, relevant to present conditions and future scenarios [48].

The experimental design enabled testing the macroalgal responses to increasing *p*CO_2_ as a function of their carbonate content, in presence or absence of grazers, and the sensitivity of sea urchins to ocean acidification, through direct and diet-mediated effects, in order to assess whether low pH conditions may lead to different grazing capabilities, as a function of their requirement for carbonate ions to build-up their carbonate structures, and to different levels of defence to predation. Another novelty of the present study is the focus on the juvenile urchin stage, whose morphology and feeding behaviour are the same of adults, but whose growth rate (skeletons and jaws) is faster and purportedly more sensitive to pH decrease [49]. Given their faster growth rate, their responses (in terms *e.g.* of jaw and test growth) under short-term experimental conditions may better be assessed.

## Materials and Methods

### Experimental set-up

Juveniles *of Paracentrotus lividus*, about 4 months old, were provided by a sea urchin hatchery in Camogli (NW Mediterranean Sea, Italy), where they had been reared after *in vitro* fertilization. A total of 144 juveniles, with a test diameter of 5 to 6 mm, had been randomly selected and moved to the Laboratoire d'Océanographie de Villefranche, where the experiment was performed, one month before the start of the experiment. Algal specimens were collected in the Bay of Villefranche (NW Mediterranean Sea, France) at a depth of less than 5 m, and transported to Villefranche laboratory. No specific permits were required for collecting specimens in the present location: the Bay of Villefranche is not subject to particular protection restrictions. The location is not privately-owned or protected in any way. For each of the 3 selected algal species, *Corallina elongata, Cystoseira amentacea* and *Dictyota dichotoma*, 32 samples of around 5 g fresh weight (FW) were collected, cleaned of epiphytes, fastened with a rubber band and placed in experimental aquaria. The field collection did not involve endangered or protected species. All algal specimens were acclimated in a thermostated room at 22°C, for at least one week before the start of the experiment: the relatively short acclimation period was due to the decision of not adding nutrients for the macroalgal culture. We decided not to add any culture medium to the tanks in order to avoid possible biases due to fact that nutrient enrichment and pH interplay in different directions on growth of non-calcifying and coralline algae [23] and could possibly exert negative effects on juvenile urchins.

Four *p*CO_2_ levels, chosen according to best practices [50] and IPCC projections [48], were used: (1) present day, *p*CO_2_  =  390 µatm (control), (2) optimistic scenario, *p*CO_2_  =  550 µatm, (3) realistic scenario (close to what expected for 2100), *p*CO_2_  =  750 µatm and (4) pessimistic scenario, *p*CO_2_  =  1000 µatm.

Unfiltered seawater, pumped from a depth of 10 m in the Bay of Villefranche, was continuously supplied to four 200 l header tanks. The chosen *p*CO_2_ levels in the experimental tanks were obtained by bubbling pure-CO_2_ using a continuous pH-stat system (IKS, Karlsbad, Aquastar). In the control tank, *p*CO_2_ was maintained at 390 µatm by bubbling CO_2_-free air produced by stripping CO_2_ from ambient using soda lime, and adjusting to the appropriate level through the IKS system. pH values corresponding to each *p*CO_2_ level were estimated based on desired *p*CO_2_, total alkalinity (*A*_T_), temperature and salinity using the R package seacarb [51]. pH electrodes from the pH-stat system were inter-calibrated every 2 days using a glass combination electrode (Metrohm, electrode plus) calibrated on the total scale using TRIS buffer solutions with a salinity of 35 [52].

**Figure 1:**
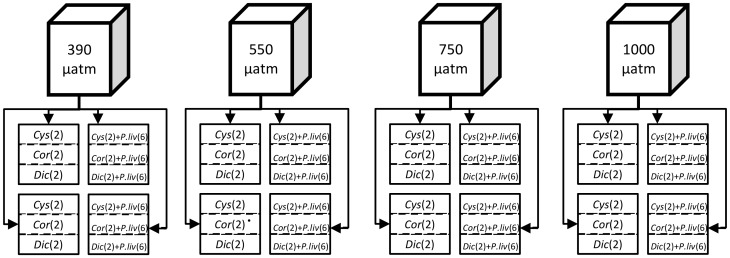
Scheme of the experimental set-up. The top boxes represent the four reservoirs in which the *p*CO_2_ was regulated. Squares represent the 16 experimental aquaria, divided into sub-sections as described in the Methods, to yield a total of 48 independent experimental units. The numbers of samples of each macroalgal item and of *P. lividus* are reported in brackets.

The whole system comprised 48 experimental units allocated in 16 aquaria (20 l), 4 aquaria for each *p*CO_2_ level (Fig. 1). Three smaller containers, each representing one experimental unit, were placed in each aquarium. Manipulated seawater from the four header tanks was delivered to experimental units at a rate of about 6 l h^−1^. Each experimental unit was directly provided inflow water from the respective reservoir at any given pH with an individual pipe. The water filled experimental units and flowed out of the smaller containers, filling the aquarium, then was discharged through an overflow system. For each *p*CO_2_ level, two aquaria (6 experimental units) with only algae and two aquaria (6 experimental units) with algae and sea urchins were set up. The design enabled us to tease apart weight loss due to sea urchin grazing and direct *p*CO_2_ effects on algal species. One of the three different algal species was allocated to each of the three experimental units in each aquarium (Fig. 1); two 5 g samples of each species were placed in their respective units. In the dedicated aquaria, six juvenile urchins were placed in each experimental unit. The experimental units within each aquarium were covered with a net in order to prevent urchin movement from one section to another, and to force them to graze on a single algal species, but with mesh large enough not to reduce light and water flow.

Irradiance values in the aquarium were maintained at about 215 µmol photons m^−2^s^−1^, corresponding to the irradiance at ca. 5 m depth in the Bay of Villefranche in June-July [19]. Light was supplied by two 39 W fluorescent tubes (JBL Solar Ultra Marin Day), with reflectors, above each aquarium (at a distance of ca. 10 cm from the water surface), with a 12∶12 h L:D photoperiod.

### Carbonate chemistry and specimens measurements

Seawater samples for total alkalinity (*A*_T_) measurements were collected twice a week, filtered on GF/F membranes and immediately analyzed. *A*_T_ was determined potentiometrically using a Metrohm titrator (Titrando 80) and a glass electrode (Metrohm, electrode plus) calibrated using first NBS buffers (pH 4.0 and pH 7.0, to check that the slope was Nernstian) and then using TRIS buffer solutions. Triplicate titrations were performed on 50 ml sub-samples at 25°C and *A*_T_ was calculated as described by Dickson *et al.* (2007). Titrations of standard seawater provided by A.G. Dickson (batch 106) yielded *A*_T_ values within 2.4 µmol kg^−1^ of the nominal value (standard deviation  =  4.6 µmol kg^−1^). All parameters of the carbonate chemistry were determined from pH_T_, *A*_T_, temperature and salinity using the R package seacarb [51].

Algae and urchins wet weights were measured at the beginning and at the end of the experiment (precision: 0.001 g). At the end of the experiment, all specimens were air dried and stored for following analyses. Macroalgal weight loss under experimental conditions was calculated as the difference between algal wet weight at the beginning and at the end of the experiment.

A subset of 24 sea urchins (one for each experimental unit) was dissected and immersed in 1% (w:v) sodium hypochlorite for 30 min in order to remove organic matter and facilitate the separation of structural elements. The length of all portions of Aristotle's lantern and the diameter of the test were measured under a stereomicroscope and the ratio between the length of the jaw pyramids of the Aristotle's lantern and the diameter of the test was calculated (hereafter referred as jaw/test ratio).

Test robustness was measured on another sub-set of 24 sea urchin specimens using a custom-made device designed to measure the static force required to crush sea urchin tests (adapted from [53]). Sea urchins were positioned upside down (in order to mimic fish predator attack) in a glass column. Then, a hollow piston, built to fit and run within the column, was inserted inside the column and progressively filled with lead pellets in order to increase the pressure, until the crushing of the urchin test. The static force required to crush sea urchin tests was measured as the weight (g) of piston and lead added: data were normalized by the diameter of the test.

Finally, scanning electron microscopy (SEM) images of different parts of sea urchins (apical disc, spines and jaw pyramids) were taken using an Environmental Scanning Electron Microscope (E-SEM VEGA3, TESCAN) at the University of Genoa.

### Statistical analyses

The effect of *p*CO_2_ on calcifying (*C. elongata*) and non-calcifying (*C. amentacea*, *D. dichotoma*) algae in presence or absence of *P. lividus* on the percent algal weight loss (arcsin transformed) was tested using a 3-way crossed ANOVA, after test for normality and homogeneity of variance: factor 1 “algal species”, 3 levels (*C. elongata*, *C. amentacea*, *D. dichotoma*); factor 2 “*p*CO_2_”, 4 levels (*p*CO_2_  =  390, 550, 750, 1000 µatm); factor 3 “urchins”, 2 levels (absent/present). Replicate values (n = 2) were the mean weight losses of the two samples of algae in each experimental unit. Student–Newman–Keuls (SNK) tests were performed for the *a posteriori* comparisons of means to check for differences among factor levels when the ANOVA detected significant effects.

Then, 2-way crossed ANOVA and SNK tests was performed on untransformed data to assess potential changes in the jaw/test ratio of the sea urchins fed with the three macroalgae and exposed to the four different pH levels. Considered factor were: factor 1 “algal species”, 3 levels (*C. elongata*, *C. amentacea*, *D. dichotoma*); factor 2 “*p*CO_2_”, 4 levels (*p*CO_2_  =  390, 550, 750, 1000 µatm). Replicate values (n = 2) were the mean values derived from urchins investigated in each independent experimental unit. A 2-way ANOVA, testing the same factors, and SNK tests were performed also on untransformed data of the normalised weight required to crush the test, in order to detect potential differences in test robustness among urchins fed with different algae and kept at different *p*CO_2_ levels.

## Results

Parameters of the carbonate chemistry are reported in Table 1. pH_T_ was maintained at an average (±SD) of (1) 8.09 ± 0.04, (2) 7.98 ± 0.06, (3) 7.84 ± 0.04 and (4) 7.70 ± 0.03, in the four treatments, respectively. *A*_T_ levels remained stable across treatments and during the whole experiment.

**Table 1:** Parameters of the carbonate system and temperature in each treatment (mean ± SD).

Treat	pH_T_	pCO_2_ (µatm)	*A*_T_ (mmol kg^−1^)	CO_3_^2−^ (mmol kg^−1^)	HCO_3_^−^ (mmol kg-1)	*C*_T_ (mmol kg-1)	Ω_calcite_	Ω_aragonite_	T(°C)
T1	8.09 ± 0.04	382 ± 41	2.531 ± 0.005	0.260 ± 0.023	1.891 ± 0.059	2.162 ± 0.037	6.10 ± 0.55	4.023 ± 0.378	24.18 ±1.12
T2	7.98 ± 0.06	528 ± 87	2.530 ± 0.005	0.215 ± 0.022	2.002 ± 0.055	2.232 ± 0.036	5.03 ± 0.52	3.322 ± 0.344	24.40 ±1.30
T3	7.84 ± 0.04	755 ± 87	2.530 ± 0.006	0.167 ± 0.017	2.121 ± 0.039	2.309 ± 0.025	3.91 ± 0.40	2.581 ± 0.268	24.32 ±1.08
T4	7.70 ± 0.03	1093 ± 72	2.530 ± 0.005	0.126 ± 0.010	2.222 ± 0.019	2.378 ± 0.012	2.95 ± 0.23	1.947 ± 0.154	24.33 ±0.97

**Figure 2:**
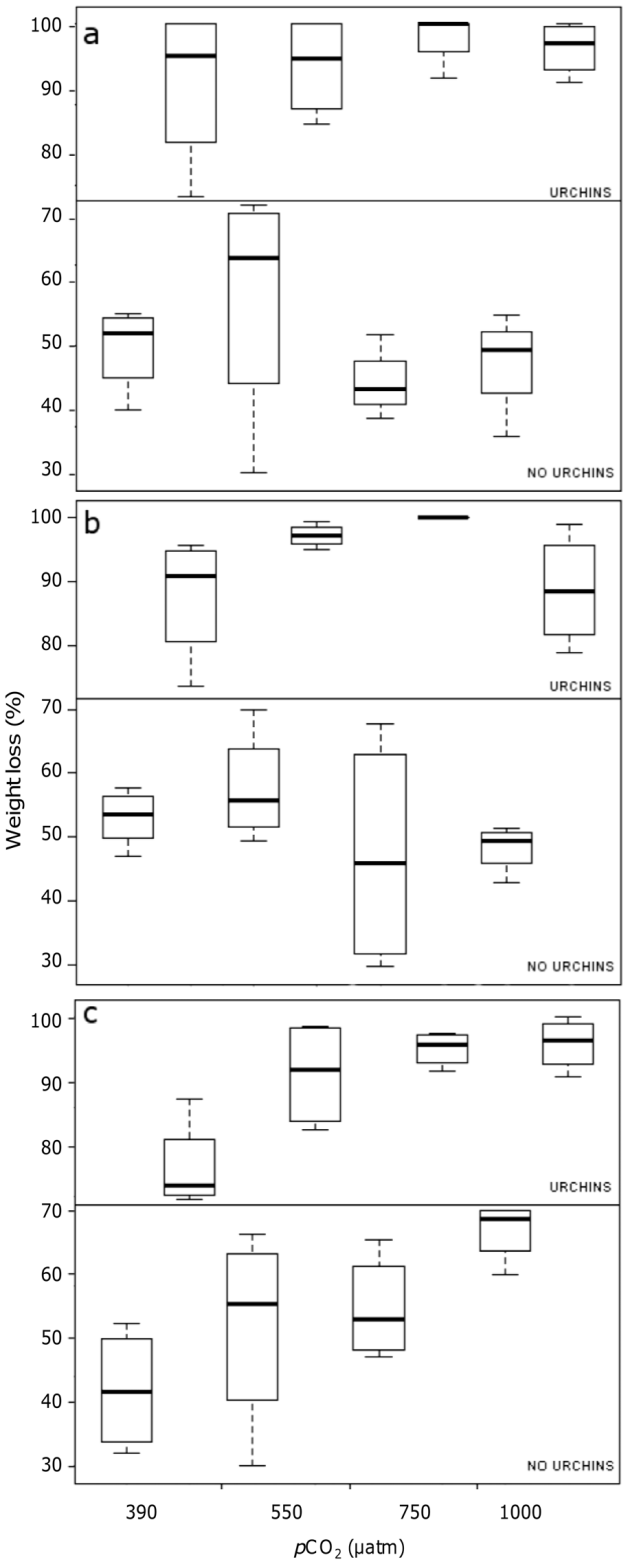
Box plot on percent weight loss as a function of *p*CO_2_ (µatm), for each macroalga: (a) *Cystoseira amentacea*, (b) *Dictyota dichotoma* and (c) *Corallina elongata.* Medians are highlighted in bold; bars represent the 25% and 75% quartiles; whiskers represent the lowest and highest data points.

Although irradiance and temperature in the experimental aquaria were chosen in order to mimic natural conditions, no growth of algal thalli was observed during the experiment in any of the treatments. Accordingly, all algal specimens showed a general decrease in weight in all treatments, including in the experimental units without sea urchins. As expected, weight loss was much larger in the units where urchins were present and represented between 65 and 100% of the amount of algae placed in the aquaria at the beginning of the experiment. In general, *C. amentacea* and *D. dichotoma* showed larger weight loss than *C. elongata*, irrespective of the *p*CO_2_ level: across all pH treatments *C. amentacea* lost around 90–100% of its weight when grazed by urchins and 55–65% when not grazed (Fig. 2a). *D. dichotoma* lost around 85–100% in presence of urchins and 45–55% when urchins were absent (Fig. 2b). *C. elongata*, probably more tolerant to artificial conditions, lost noticeably less weight than the other two algae in the treatments without urchins: around 40% in the control, till 60% at the highest *p*CO_2_ treatment (Fig 2c). Yet, weight loss of *C. elongata* increased with increasing *p*CO_2_, both in the presence (from 75 to 95%) and absence of sea urchins (Fig. 2c). The statistical significance of these results is shown in Table 2 which highlighted a significant effect of the “algal spp.” and “*p*CO_2_” interaction (p<0.05) and of the factor “urchins” (*p*<0.001) on all algal species. The SNK test on the factor “urchins” confirmed the expected larger weight loss when urchins were present at all *p*CO_2_ levels (presence > absence; *p*<0.01). For the interaction between factors “algal spp.” and “*p*CO_2_”, SNK test revealed a significantly larger weight loss of *C. elongata* at elevated *p*CO_2_ treatments than at the control conditions (*p*CO_2_ (550 = 750 = 1000) > 390 µatm; *p*<0.01; Table 2), while no differences were observed for the two non calcifying species.

**Table 2:** Three-way ANOVA on algal weight loss.

Source	SS	DF	MS	F	P
algal species	1493.56	2	746.78	17.65	0.0000
*p*CO_2_	655.71	3	218.57	5.16	0.0068
Urchins	12076.74	1	12076.74	285.38	**0.0000**
algal spp. X *p*CO_2_	726.15	6	121.02	2.86	**0.0302**
algal spp. X urchins	63.24	2	31.62	0.75	0.4844
*p*CO_2_ X urchins	337.50	3	112.50	2.66	0.0711
algal spp. X *p*CO_2_ X urchins	167.34	6	27.89	0.66	0.6829
Residuals	1015.62	24	42.32		
Total	16535.87	47			

SNK tests:

Algal species (pCO_2_): at 390 µatm C. amentacea  =  D. dichotoma > C. elongata (p<0.01); at 550 µatm, C. amentacea  =  D. dichotoma > C. elongata (p<0.05); at 750 µatm: D. dichotoma > C. elongata (p<0.05); at 1000 µatm: C. amentacea  =  D. dichotoma  =  C. elongata;

*p*CO_2_ (Algal species): for *C. amentacea* and *D. dichotoma* 390 = 550 = 750 = 1000 µatm; for *C. elongata p*CO_2_ 550 = 750 = 1000 > 390 µatm (*p*<0.01);

Urchins: presence > absence (*p*<0.01).

All factors are orthogonal and fixed. Number of replicates  =  2. Cochran's test is not significant (C = 0.19). The significant effects are highlighted in bold. F values were calculated *versus* MS of residuals.

**Figure 3:**
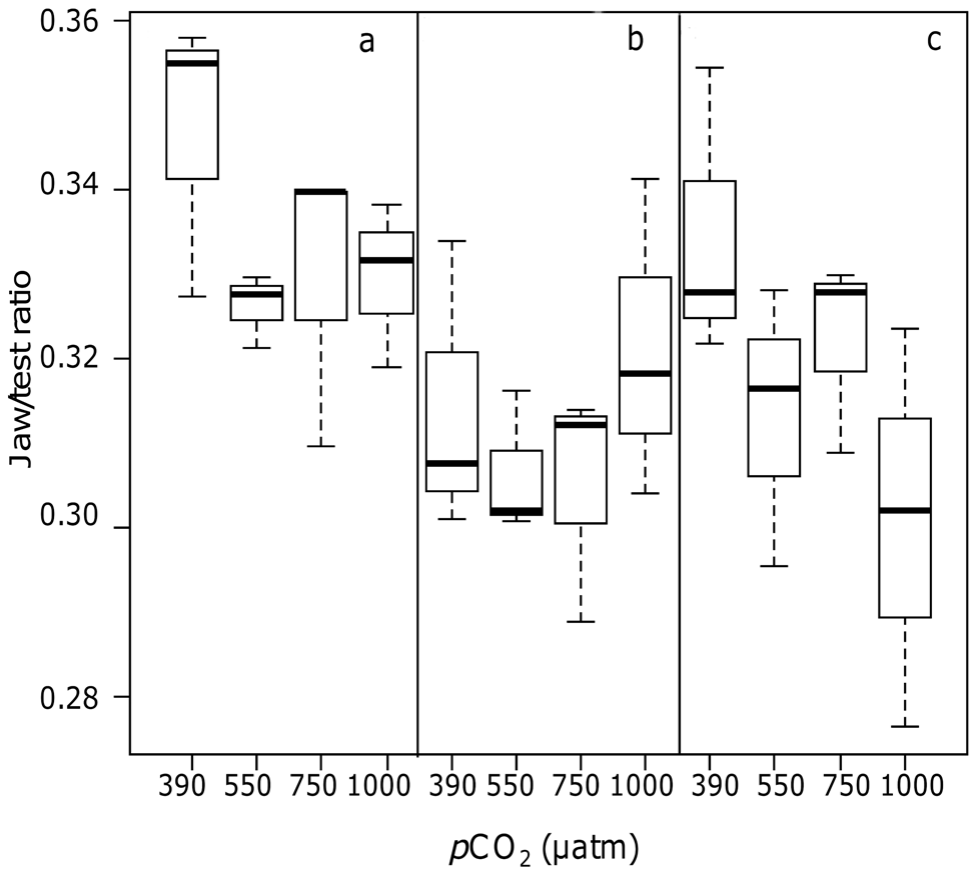
Box plot on the jaw/test ratio as a function of *p*CO_2_ and algal diet: sea urchins fed with (a) *Corallina elongata,* (b) *Cystoseira amentacea* and (c) *Dictyota dichotoma.* Medians are highlighted in bold; bars represent the 25% and 75% quartiles; whiskers represent the lowest and highest data points.

Some mortality of sea urchins, unrelated to the *p*CO_2_ level, occurred during the first week (19 out of 144 specimens, 15 of them were fed with *D. dichotoma*). The remaining ones were in very good conditions after one month, and showed positive increase in size. The jaw/test ratio showed a weak significant difference only between control *p*CO_2_ and 750 µatm (390 > 750 µatm; p<0.05) and no significant interaction between factor “algal species” and “*p*CO_2_” was observed (ANOVA; Table 3). Significant differences were found for the factor “algal species” (*p*<0.01) and the SNK test highlighted significant higher values of the ratio in urchins fed with *C. elongata*, compared to *C. amentacea* and *D. dichotoma* (*C. elongata* > (*C. amentacea*  =  *D. dichotoma*); *p*<0.01). In Figure 3, box plots show jaw/test ratio values for urchins fed with the three different algae, kept at the four different pH conditions.

**Table 3:** Two-way ANOVA on the jaw/test ratio.

Source	SS	DF	MS	F	P
algal species	0.0036	2	0.0018	10.13	**0.0027**
*p*CO_2_	0.0019	3	0.0006	3.61	**0.0458**
algal spp. X *p*CO_2_	0.0024	6	0.0004	2.20	0.1157
Residuals	0.0021	12	0.0002		
Total	0.0100	23			

SNK tests:

Algal species: *C. elongata* > (*C. amentacea*  =  *D. dichotoma*) (*p*<0.01);

*p*CO_2_: 390 > 750 µatm (p<0.05).

All factors are orthogonal and fixed. Number of replicates  =  2. Cochran's Test is not significant (C = 0.22). The significant effects are highlighted in bold. F values were calculated *versus* MS of residuals.

**Figure 4:**
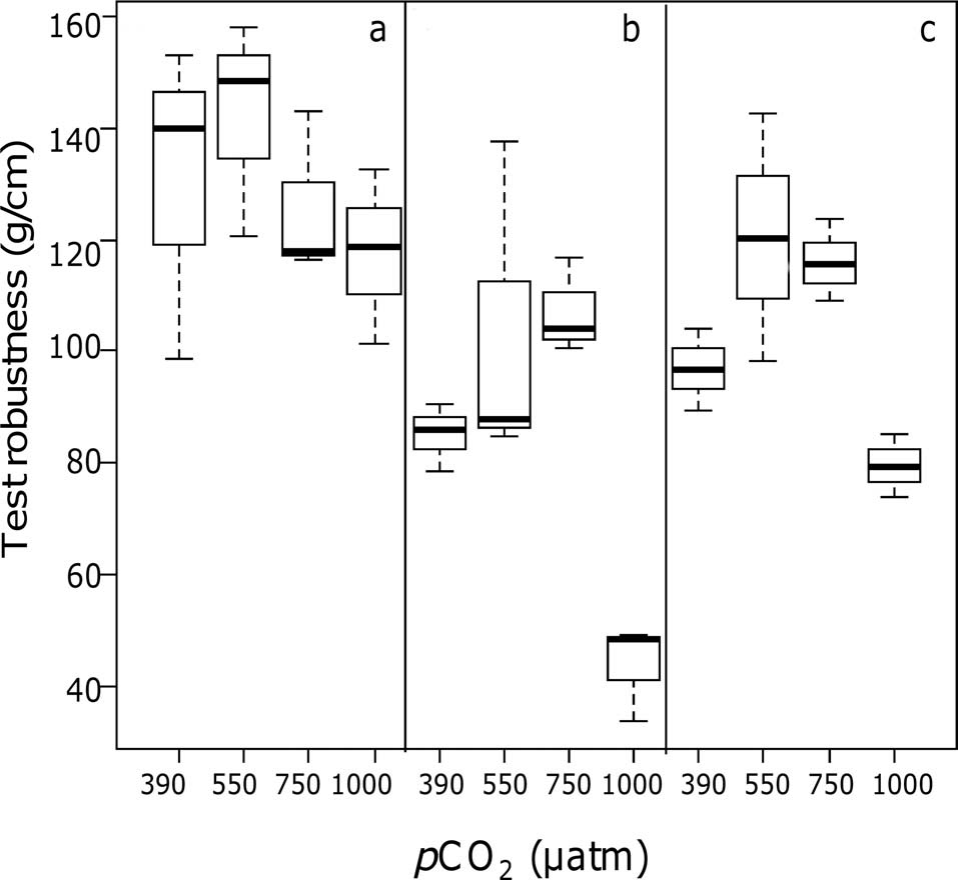
Box plot on test robustness, expressed as the weight (g) needed to crush the urchin test normalized by the test diameter, as a function of *p*CO_2_ and algal diet: sea urchins fed with (a) *Corallina elongata*, (b) *Cystoseira amentacea* and (c) *Dictyota dichotoma.* Medians are highlighted in bold; bars represent the 25% and 75% quartiles; whiskers represent the lowest and highest data points.

**Figure 5:**
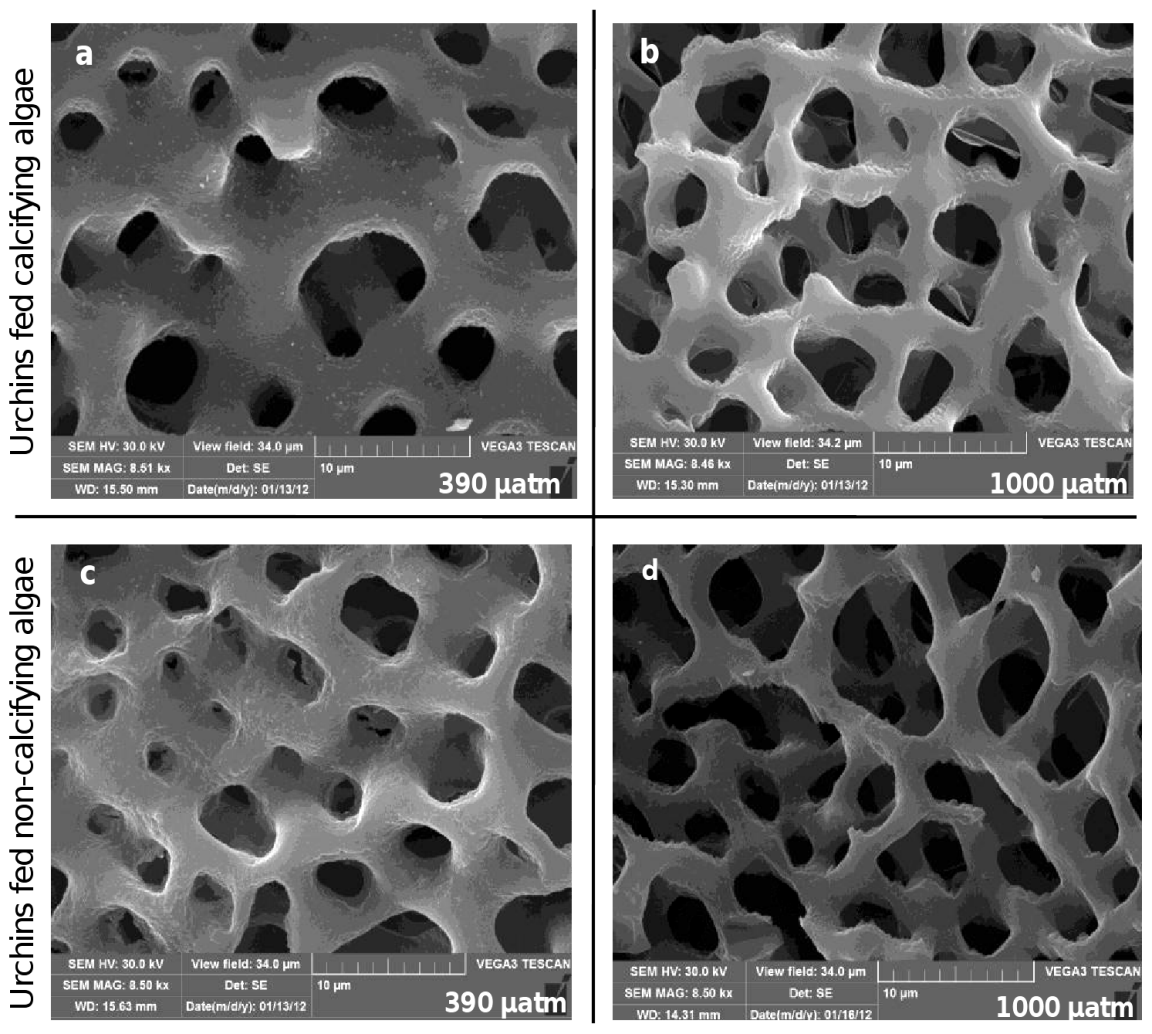
SEM images (8500x) of portions of the Aristotle's lantern of urchins fed calcifying (*C. elongata*; a, b) and non-calcifying (*C. amentacea*; c, d) macroalgae and maintained at *p*CO_2_ levels of 390 (a, c) and 1000 µatm (b, d) for 1 month.

The effects of *p*CO_2_ and algal species on test robustness (Fig. 4) were both significant (ANOVA, *p*<0.05; Table 4). Test robustness was significantly lower in urchins kept at the highest *p*CO_2_ level (*p*CO_2_ (390 = 550 = 750) > 1000 µatm; *p*<0.05) and changed according to the diet, across all pH treatments: tests of urchins fed with *C. elongata* were significantly stronger compared to urchins fed with the two non-calcifying species (*C. elongata* > (*C. amentacea*  =  *D. dichotoma*)). Scanning Electron Microscope images performed on the apical disc, spines and, Aristotle's lantern of sea urchins showed that only the Aristotle's lantern was affected by the experimental treatments, while the other portions investigated did not reveal clearly detectable differences. The surface of latero-radial sides of the Aristotle's lantern, usually characterized by fine and dense tridimensional mesh of calcite trabeculae [54], examined with SEM at a magnification of 8500x, showed a porous structure with larger and more irregularly shaped holes, increasing signs of corrosion and structural breaks as a function of increasing *p*CO_2_ (Fig. 5). The porous structure of Aristotle's lantern of urchins fed with *C. elongata* at 390 µatm (Fig. 5a) looked denser and Aristotle's lantern surface was smoother than those of urchins fed with the same diet but maintained at elevated *p*CO_2_ (*e.g.* 1000 µatm, Fig. 5b) and also to those of urchins fed with non-calcifying macroalgae (*e.g.* urchins fed with *C. amentacea*, Fig. 5c) at the same *p*CO_2_ (390 µatm). The density and preservation of the trabecular structure of the Aristotle's lantern of urchins that grazed on non-calcifying macroalgae exhibited a larger damage at elevated *p*CO_2_ (Fig. 5d).

**Table 4:** Two-way ANOVA on test robustness.

Source	SS	DF	MS	F	P
algal species	6164.02	2	3082.01	6.55	**0.0119**
*p*CO_2_	6253.78	3	2084.59	4.43	**0.0257**
algal spp. X *p*CO_2_	1367.76	6	227.96	0.48	0.8078
Residuals	5645.56	12	470.46		
Total	19431.13	23			

SNK tests:

Algal species: *C. elongata* > (*C. amentacea*  =  *D. dichotoma*) (*p*<0.05);

*p*CO_2_: (390 = 550 = 750) > 1000 µatm (p<0.05).

All factors are orthogonal and fixed. Number of replicates  =  2. Cochran's Test is not significant (C = 0.26). The significant effects are highlighted in bold. F values were calculated *versus* MS of residuals.

## Discussion

### Effects of ocean acidification and grazing on calcifying and non-calcifying macroalgae

The role that ocean acidification could play on macroalgal assemblages, potentially affecting different processes, such as photosynthesis, growth, calcification rate and competitive ability of macroalgae, depending on their carbonate content and deposition pathway, is difficult to unravel. Macroalgae rely for photosynthesis on CO_2_ and/or HCO_3_^−^ [55] which will increase in the ocean acidification scenario, potentially stimulating primary production [56]. Yet, the decrease in pH reduces CO_3_^2−^, which is used by calcifying macroalgae for the production and maintenance of carbonate structures [57]–[59], reducing growth [23], [25] and calcification [28]–[29]. Additionally, calcifying species may also be negatively affected by increased CO_2_ indirectly, as a consequence of the increased competitive ability of non-calcifying macroalgae [60].

The present study confirms previous findings, both based on *in situ* observations [18], [30] and laboratory experiments [19]–[20], [23], [60], which have shown different responses of calcifying and non-calcifying macroalgae to ocean acidification.

The weight loss of the non-calcifying species, *Cystoseira amentacea* and *Dictyota dichotoma* was unaffected by *p*CO_2_. In contrast, the calcifying seaweed *Corallina elongata* showed a significantly larger weight loss in the three elevated *p*CO_2_ treatments than in the control condition. The sensitivity of coralline algae to elevated *p*CO_2_ has already been reported by previous studies, some of which actually contrasted the response to nowadays *p*CO_2_ conditions compared to *p*CO_2_ levels higher than those projected in the incoming decades (ranging between 700 and 1500 µatm; *e.g.* [7], [19], [25], [29], [61]). The present study, testing *p*CO_2_ levels in the range projected in the coming century, demonstrates that *Corallina elongata* exhibits a negative response even at a relatively low *p*CO_2_ level (550 µatm), suggesting a significant impact in a few decades.

In the presence of urchins, all macroalgal species lost significantly more weight at any *p*CO_2_ compared to experimental units where urchins were absent. More interestingly, the presence of urchins increased algal weight loss at any elevate *p*CO_2_ compared to controls. The apparently smaller weight loss of *C. elongata* in the grazed condition (Fig. 2), compared to the non-calcifying species, was actually due to the lower weight loss of the calcified species also under the un-grazed condition. These findings suggest that urchin grazing pressure will be unaffected in an acidified scenario, and, as a consequence, grazing activity is expected to exacerbate *p*CO_2_ effects on macroalgae, particularly calcifying ones.

All algal species showed a weight loss during the experiment. It did not prevent testing the effects of the main factors (*p*CO_2_ and presence/absence of urchins). An increase in growth of non-calcifying species at elevated *p*CO_2_ was anticipated based on previous studies [21]–[22], [23], [62]–[63], which would suggest that photosynthesis of certain benthic autotrophs will increase in a high-CO_2_ world, except when other constituents such as nitrogen, phosphorus, and iron are limiting [8], [64]. The weight loss observed in the present study may be explained by nutrient limitation and/or artificial conditions of the experiment (*e.g*. light). Additionally, in the Mediterranean Sea, the macroalgal biomass is highest in the spring; growth is much lower in June-July, the time at which the experiment was performed.

### Combined effect of ocean acidification and diet on sea urchins

Sea urchins are the major grazers on rocky subtidal habitats [33] and their carbonate structures are particularly susceptible to pH reductions across their different developmental stages (larval skeletal rods, adult test, teeth and spines; [35], [65]–[66]).

Few studies have focused on the juvenile stage, which is potentially one of the most susceptible to acidified conditions, given their higher growth rate compared to adults. In fact, even a small change in *p*CO_2_ (200 µatm) is reported to increase mortality, decrease growth and cause a thinner and more easily breakable test in one year old urchins [49].

The present study shows significant *p*CO_2_ effects on test robustness: sea urchins maintained at the lower pH (pH_T_ 7.7) exhibited a less robust test than the ones maintained at higher pH. Yet, lower test robustness, *per se*, did not affect mortality during the short experimental time. In addition to direct *p*CO_2_ effects, our study points out that the effect of ocean acidification on juveniles of *Paracentrotus lividus* is mediated by their diet, both concerning test robustness and jaw size, potentially affecting grazing capacity. The food source caused differences in the robustness of the test: urchins fed with *C. elongata* displayed a significantly stronger test than the ones fed with the two non-calcifying species.

These findings (the threshold of pH_T_ 7.7 in sea urchin sensitivity and the role of coralline in their diet for strengthening the test) are in agreement with the findings of Hall-Spencer *et al*. (2008) in naturally acidified areas: a threshold value of mean pH_T_ 7.8 is reported below which sea urchins are absent; accordingly, in these pH_T_ conditions coralline algae are missing. Combining the results of these two studies suggests that a pH_T_ value of 7.7–7.8 and the lack of corallines in urchin diet does not increase directly the sea urchin mortality but, rather, makes them more susceptible to predation, because of the less robust tests.

Additional diet related differences were observed in the jaw/test ratio, a parameter notoriously variable according to the trophic condition of the individual [67]–[69]. Higher values of this index, that means larger lantern compared to the test size, were found in urchins fed with *C. elongata* than in urchins fed with the other species, irrespectively of the *p*CO_2_ level. In Figure 3 appear that *p*CO_2_ modulates the diet effects as the ratio in sea urchins fed with *C. elongata* was higher at 390 µatm than at the three more elevated *p*CO_2_ levels. Sea urchins fed with *D. dichotoma* only exhibited a clear difference between the control and the highest *p*CO_2_ treatment (1000 µatm) (Fig. 3c). No difference among *p*CO_2_ treatments was observed in specimens fed with *C. amentacea* (Fig. 3b). The different response to the three algal species, additionally modulated by *p*CO_2_ in the calcifying species (ratio higher in urchins fed with *C. elongata* in the control condition, and lower with increasing *p*CO_2_ and in the urchins fed with the non-calcifying algae), may be due to the different toughness of the algal tissues. It is hypothesized that sea urchins need a larger grazing apparatus (relative to the body size) when fed with more calcified algal structures: in *Corallina*-fed urchins the ratio decreases sharply from the controls to the first intermediate *p*CO_2_ level, showing a fast response to *C. elongata* decalcification, that makes the alga more easily grazable [27].

Scanning Electron Microscope images on latero-radial sides of Aristotle's lantern highlighted, for all sea urchins exposed to experimental conditions, a progressive weakening of the calcite trabecular structure as a function of increasing *p*CO_2_. While the Aristotle's lantern of urchins fed with *C. amentacea* and *D. dichotoma* looked generally less tough in all treatments, *Corallina*-fed urchins displayed denser and smoother structures in the controls compared to controls fed with the other algae, with a clear reduction in thickness and increase in corrosion as a function of increasing *p*CO_2_.

Taken together, these results suggest that the uptake of carbonate from the diet is fundamental in modulating sea urchin response to ocean acidification. The carbonate content of *C. elongata*, even at the higher *p*CO_2_, makes the test of the juveniles much stronger and their jaws larger compared with specimens fed with non-calcifying algae. These findings, that show a direct *p*CO_2_ effect on sea urchin defense from predation (test robustness) but also strong indirect effects mediated by the diet on test robustness and on jaw size, were achieved thanks to the experimental setup of an artificial community, designed for assessing interactions among primary producers and their grazers.

### Cascading effects of ocean acidification on prey-predator dynamics

Ocean acidification, whether causing the loss of keystone/critical species or the reduction in their activity (*e.g.* predation, grazing, bioturbation), could have consequences at the ecosystem level [70], for example reducing habitat complexity and interfering with biological controls, such as the top-down control of sea urchins on non-calcifying macroalgae [44]. Our results highlight that in the different combinations of *p*CO_2_ and diet, sea urchins showed different morpho-functional features, more as a consequence of diet than of *p*CO_2_. Notwithstanding these differences, the grazing pressure of the urchins was similar across all the treatments. The observed combined effects of ocean acidification and macroalgal diet on test robustness and on jaw/test ratio may have severe consequences at the ecosystem level. The decrease in test robustness due to the diet and *p*CO_2_ is anticipated to make juveniles of *P. lividus* more susceptible to predation by fish, *e.g. Diplodus* spp. [71]–[72], because the decreased robustness of sea urchin skeletons makes them less resistant to static loads whenever they are attacked by crushing predators [53].

The consequent reduced sea urchin density is expected to cause a reduction of their grazing pressure on macroalgal assemblages, with different effects according to the state of the habitat. In barren ground habitats, the reduced abundance of sea urchins would favor the recolonisation of non-calcifying macroalgae [73], potentially causing an increase in macrobenthic biomass and biodiversity. Conversely, in well vegetated, undisturbed environments, biodiversity may be negatively affected by i) the loss of coralline species, ii) the reduced number of colonizable patches produced by sea urchins grazing [34], iii) the lack of the succession promoting role of coralline algae which positively affects recovery and complexity in rocky reef communities after disturbance ([74]–[77], authors unpublished data). Consequently, the predicted effects of ocean acidification will lead to a decrease in biodiversity in well vegetated, undisturbed environments, while, considering the interaction with cumulated human impacts [78]–[79], particularly overfishing (favoring barren grounds formation), non-additive antagonistic effects on macroalgal biomass are expected. The proliferation of sea-urchins due to overfishing will be mitigated in a ocean acidification scenario, also due to higher vulnerability to predation, as shown in this study, favoring the recovery from barren grounds to well vegetated assemblages. The barren recovery trajectory is expected to lead to an increase of macroalgal biomass, but this may be locally unpredictable [80], depending on a multitude of biotic and abiotic factors (*e.g*. abundance of other grazers, extent of barren area and distance from well vegetated areas, availability of propagules, presence of other human impacts).

The recovery of foundation species (*e.g. Cystoseira*), in absence of coralline macroalgae, will be potentially prevented by the installation of turfs, able to inhibit their recruitment and known to be promoted in the framework of cumulated human impacts (see [81]).

These findings stress the need to move from experiments on individual species to species interactions in order to better understand the both direct and indirect effects driven by top-down and bottom-up processes, in order to build more reliable predictions of future scenarios under the interaction of high *p*CO_2_ conditions and cumulated human impacts.

## References

[1] Keeling CD, Piper SC, Bollenbacher AF, Walker JS (2009) Atmospheric CO_2_ records from sites in the SIO air sampling network. Trends: A compendium of data on global change. Carbon Dioxide Information Analysis Center, Oak Ridge National Laboratory, U.S. Department of Energy, Oak Ridge, Tenn., USA.

[2] Gattuso J-P & Hansson L (2011) Ocean acidification: background and history. In: Gattuso J-P, Hansson L, editors. Ocean Acidification. Oxford University Press, Oxford. pp. 1–20.

[3] Orr JC (2011) Recent and future changes in ocean carbonate chemistry. In: Gattuso J-P, Hansson L, editors. Ocean Acidification. Oxford University Press, Oxford. pp. 41–66.

[4] Pörtner HO, Gutowska M, Ishimatsu A, Lucassen M, Melzner F et al.. (2011) Effects of ocean acidification on nektonic organisms. In: Gattuso J-P, Hansson L, editors. Ocean Acidification. Oxford University Press, Oxford.

[5] Gattuso JP, Allemand D, Frankignoulle M (1999) Photosynthesis and calcification at cellular, organismal and community levels in coral reefs: a review on interactions and control by carbonate chemistry. Am Zool 39 (1): 160–183.

[6] Kleypas JA & Langdon C (2006) Coral reefs and changing seawater carbonate chemistry. Coastal and estuarine studies 61: 73–110.

[7] Anthony K, Kline D, Diaz-Pulido G, Dove S, Hoegh-Guldberg O (2008) Ocean acidification causes bleaching and productivity loss in coral reef builders. Proc Natl Acad Sci USA 105 (45): 17442. 10.1073/pnas.0804478105 PMC2580748 18988740

[8] Andersson AJ, Mackenzie FT, Gattuso JP (2011) Effects of ocean acidification on benthic processes, organisms, and ecosystems. In: Gattuso J-P, Hansson L, editors. Ocean Acidification. Oxford University Press, Oxford. pp. 122–153.

[9] Nelson WA (2009) Calcified macroalgae critical to coastal ecosystems and vulnerable to change: A review. Mar Freshw Res 60(8): 787–801.

[10] Ballesteros E, Torras X, Pinedo S, Garcia M, Mangialajo L, et al (2007) A new methodology based on littoral community cartography dominated by macroalgae for the implementation of the European Water Framework Directive. Mar Pollut Bull 55(1–6): 172–180. 17045303 10.1016/j.marpolbul.2006.08.038

[11] Mangialajo L, Ruggieri N, Asnaghi V, Chiantore M, Povero P, et al (2007) Ecological status in the Ligurian Sea: The effect of coastline urbanisation and the importance of proper reference sites. MarPollut Bull 55(1–6): 30–41. 10.1016/j.marpolbul.2006.08.022 17010997

[12] Asnaghi V, Chiantore M, Bertolotto R, Parravicini V, Cattaneo-Vietti R, et al (2009) Implementation of the European Water Framework Directive: natural variability associated to the CARLIT method on the rocky shores of the Ligurian Sea (Italy). Marine Ecology 30: 505–513.

[13] Sala E, Boudouresque CF, Harmelin-Vivien M (1998) Fishing, Trophic Cascades, and the Structure of Algal Assemblages: Evaluation of an Old but Untested Paradigm. Oikos 82(3): 425–439.

[14] Airoldi L (2003) The effects of sedimentation on rocky coast assemblages. Oceanography and Marine Biology: an Annual Review 41: 161–236.

[15] Connell SD (2005) Assembly and maintenance of subtidal habitat heterogeneity: synergistic effects of light penetration and sedimentation. Mar Ecol Prog Ser 289 53–61.

[16] Mangialajo L, Chiantore M (2008) Cattaneo-Vietti R (2008) Loss of fucoid algae along a gradient of urbanisation, and structure of benthic assemblages. Mar Ecol Prog Ser 358: 63–74.

[17] Guidetti P & Dulçic J (2007) Relationships among predatory fish, sea urchins and barrens in Mediterranean rocky reefs across a latitudinal gradient. Mar Environ Res 63: 168–184. 17034843 10.1016/j.marenvres.2006.08.002

[18] Hall-Spencer JM, Rodolfo-Metalpa R, Martin S, Ransome E, Fine M, et al (2008) Volcanic carbon dioxide vents show ecosystem effects of ocean acidification. Nature 454(7200): 96–99. 18536730 10.1038/nature07051

[19] Martin S & Gattuso J-P (2009) Response of Mediterranean coralline algae to ocean acidification and elevated temperature. Glob Change Biol 15: 2089–2100.

[20] Connell SD & Russell BD (2010) The direct effects of increasing CO_2_ and temperature on non-calcifying organisms: increasing the potential for phase shifts in kelp forests. Proc R Soc B-Biol Sci 277(1686): 1409–1415. 10.1098/rspb.2009.2069 PMC2871943 20053651

[21] Kübler JE, Johnston AM, Raven JA (1999) The effects of reduced and elevated CO_2_ and O_2_ on the seaweed *Lomentaria articulata* . Plant Cell Environ 22(10): 1303–1310.

[22] Zou D (2005) Effects of elevated atmospheric CO_2_ on growth, photosynthesis and nitrogen metabolism in the economic brown seaweed, *Hizikia fusiforme* (Sargassaceae, Phaeophyta). Aquaculture 250(3): 726–735.

[23] Russell BD, Thompson JAI, Falkenberg LJ, Connell SD (2009) Synergistic effects of climate change and local stressors: CO_2_ and nutrient-driven change in subtidal rocky habitats. Glob Change Biol 15(9): 2153–2162.

[24] Kroeker KJ, Kordas LR, Crim RN, Singh GG (2010) Meta-analysis reveals negative yet variable effects of ocean acidification on marine organisms. Ecol Lett 13(11): 1419–1434. 20958904 10.1111/j.1461-0248.2010.01518.x

[25] Kuffner IB, Andersson AJ, Jokiel PL, Rodgers KS, Mackenzie FT (2007) Decreased abundance of crustose coralline algae due to ocean acidification. Nat Geosci 1: 114–117.

[26] Jokiel P, Rodgers K, Kuffner IB, Andersson AJ, Cox E, et al (2008) Ocean acidification and calcifying reef organisms: a mesocosm investigation. Coral Reefs 27(3): 473–483.

[27] Ragazzola F, Foster LC, Form A, Anderson PSL, Hansteen TH, et al. (2012) Ocean acidification weakens the structural integrity of coralline algae. Glob Change Biol DOI: 10.1111/j.1365-2486.2012.02756.x 10.1111/j.1365-2486.2012.02756.x 24501058

[28] Smith AD & Roth AA (1979) Effect of carbon dioxide concentration on calcification in the red coralline alga *Bossiella orbigniana.* . Mar Biol 52: 217–225.

[29] Gao K, Aruga Y, Asada K, Ishihara T, Akano T, et al (1993) Calcification in the articulated coralline alga *Corallina pilulifera*, with special reference to the effect of elevated CO_2_ concentration. Mar Biol 117: 129–132.

[30] Porzio L, Buia MC, Hall-Spencer JM (2011) Effects of ocean acidification on macroalgal communities. JEMBE 400(1–2): 278–287.

[31] Wootton JT, Pfister CA, Forester JD (2008) Dynamic patterns and ecological impacts of declining ocean pH in a high-resolution multi-year dataset. Proc Natl Acad Sci USA 105: 18848–18853. 19033205 10.1073/pnas.0810079105 PMC2596240

[32] Diaz-Pulido G, Gouezo M, Tilbrook B, Dove S, Anthony KRN (2011) High CO_2_ enhances the competitive strength of seaweeds over corals. Ecol Lett 14(2): 156–162. 21155961 10.1111/j.1461-0248.2010.01565.x PMC3047711

[33] Ruitton S, Francour P, Boudouresque C (2000) Relationships between algae, benthic herbivorous invertebrates and fishes in rocky sublittoral communities of a temperate sea (Mediterranean). Estuar Coast Shelf Sci 50(2): 217–230.

[34] Coma R, Serrano E, Linares C, Ribes M, Díaz D, et al (2011) Sea urchins predation facilitates coral invasion in a marine reserve. *PLoS ONE* 6(7): e22017. 21789204 10.1371/journal.pone.0022017 PMC3138760

[35] Dupont S, Ortega-Martınez O, Thorndyke M (2010) Impact of near-future ocean acidification on echinoderms. Ecotoxicology 19: 449–462. 20130988 10.1007/s10646-010-0463-6

[36] Kurihara H & Shirayama Y (2004) Effects of increased atmospheric CO_2_ on sea urchin early development. Mar Ecol Prog Ser 274: 161–169.

[37] Byrne M, Ho M, Wong E, Soars NA, Selvakumaraswamy P, et al (2011) Unshelled abalone and corrupted urchins: Development of marine calcifiers in a Changing Ocean. Proc R Soc B-Biol Sci 278(1716): 2376–2383. 10.1098/rspb.2010.2404 PMC3119014 21177689

[38] O'Donnell MJ, Todgham AE, Sewell MA, Hammond LM, Ruggiero K, et al (2009) Ocean acidification alters skeletogenesis and gene expression in larval sea urchins. Mar Ecol Prog Ser 398: 157–171.

[39] Moulin L, Catarino AI, Claessens T (2011) Dubois P (2011) Effects of seawater acidification on early development of the intertidal sea urchin *Paracentrotus lividus* (Lamarck 1816). Mar Pollut Bull 62(1): 48–54. 20950830 10.1016/j.marpolbul.2010.09.012

[40] Stumpp M, Wren J, Melzner F, Thorndyke MC, Dupont ST (2011) CO_2_ induced seawater acidification impacts sea urchin larval development I: Elevated metabolic rates decrease scope for growth and induce developmental delay. Comparative Biochemistry and Physiology - A Molecular and Integrative Physiology 160(3): 331–340. 21742050 10.1016/j.cbpa.2011.06.022

[41] Stumpp M, Dupont S, Thorndyke MC, Melzner F (2011) CO_2_ induced seawater acidification impacts sea urchin larval development II: Gene expression patterns in pluteus larvae. Comparative Biochemistry and Physiology - A Molecular and Integrative Physiology 160(3): 320–330. 21742049 10.1016/j.cbpa.2011.06.023

[42] Martin S, Richier S, Pedrotti ML, Dupont S, Castejon C, et al (2011) Early development and molecular plasticity in the Mediterranean sea urchin *Paracentrotus lividus* exposed to CO_2_-driven acidification. J Exp Biol 214(8): 1357–1368. 21430213 10.1242/jeb.051169

[43] Kroeker KJ, Micheli F, Gambi MC, Martz TR (2011) Divergent ecosystem responses within a benthic marine community to ocean acidification. Proc Natl Acad Sci USA 108 (35): 14515–14520. 10.1073/pnas.1107789108 PMC3167536 21844331

[44] Johnson VR, Russell BD, Fabricius KE, Brownlee C, Hall-Spencer JM (2012) Temperate and tropical brown macroalgae thrive, despite decalcification, along natural CO_2_ gradients. Glob Change Biol. DOI: 10.1111/j.1365-2486.2012.02716.x 10.1111/j.1365-2486.2012.02716.x 24501057

[45] Nisumaa A, Pesant S, Bellerby RGJ, Delille B, Middelburg J, et al (2010) EPOCA/EUR-OCEANS data-mining compilation on the impacts of ocean acidification. Earth System Science Data Discussions 3: 109–130.

[46] Browman HI, Vézina AF, Hoegh-Guldberg O (2008) Effects of ocean acidification on marine ecosystems. Mar Ecol Prog Ser 373: 199–201.

[47] Russell BD & Connell SD (2012) Origins and consequences of global and local stressors: incorporating climatic and non-climatic phenomena that buffer or accelerate ecological change. Mar Biol:1–7.

[48] Intergovermental Panel on Climate Change (2007) Climate Change 2007: The Physical Science Basis. Contribution of Working Group I to the Fourth Assessment Report of the Intergovernmental Panel on Climate Change, eds Solomon S, et al. (Cambridge University Press, Cambridge, UK).

[49] Shirayama Y & Thornton H (2005) Effect of increased atmospheric CO_2_ on shallow water marine benthos. J Geophys Res 110(C9): C09S08.

[50] Barry JP, Tyrrell T, Hansson L, Gattuso J-P 2010. CO_2_ targets for ocean acidification perturbation experiments. In: Riebesell U, Fabry VJ, Hansson L, Gattuso J-P (Eds.), Guide to best practices for ocean acidification research and data reporting. Luxembourg: Publications Office of the European Union, pp. 53–66.

[51] Lavigne HG, Gattuso J-P (2010) Seacarb: seawater carbonate chemistry with R. R package version 2.3.3. Available at http://cran.r-project.org/web/packages/seacarb/index.html. Accessed November 2010.

[52] Dickson AG, Sabine CL, Christian JR, eds (2007) Guide to Best Practices for Ocean CO_2_ Measurements. PICES Special Publication 3, 191 pp. North Pacific Marine Science Organization(, Sidney, BC, Canada). Website: http://cdiac.ornl.gov/oceans/- Available at: http://cdiac.ornl.gov/oceans/Handbook_2007.html. Accessed March 2013.

[53] Guidetti P & Mori M (2005) Morpho-functional defences of Mediterranean sea urchins, *Paracentrotus lividus* and *Arbacia lixula*, against fish predators. Mar Biol 147: 797–802.

[54] Carnevali MDC, Bonasoro F, Melone G (1991) Microstructure and mechanical design in the lantern ossicles of the regular sea-urchin *Paracentrotus lividus.* A scanning electron microscope study. Ital J Zoolog 58(1): 1–42.

[55] Beardall J, Beer S, Raven J (1998) Biodiversity of marine plants in an era of climate change: some predictions based on physiological performance. Bot Marina 41(1–6): 113–124.

[56] Hurd CL, Hepburn CD, Currie KI, Raven JA, Hunter KA (2009) Testing the effects of ocean acidification on algal metabolism: considerations for experimental designs. J Phycol 45: 1236–1251. 27032579 10.1111/j.1529-8817.2009.00768.x

[57] Borowitzka MA & Larkum A (1987) Calcification in algae: mechanisms and the role of metabolism. Crit Rev Plant Sci 6(1): 1–45.

[58] De Beer D & Larkum A (2001) Photosynthesis and calcification in the calcifying algae *Halimeda discoidea* studied with microsensors. Plant Cell Environ 24(11): 1209–1217.

[59] Semesi IS, Kangwe J, Björk M (2009) Alterations in seawater pH and CO_2_ affect calcification and photosynthesis in the tropical coralline alga, *Hydrolithon* sp.(Rhodophyta). Estuar Coast Shelf Sci 84(3): 337–341.

[60] Cornwall CE, Hepburn CD, Pritchard D, Currie KI, McGraw CM, et al (2011) Carbon-use strategies in macroalgae: differential responses to lowered pH and implications for ocean acidification. J Phycol 48(1): 137–144. 27009658 10.1111/j.1529-8817.2011.01085.x

[61] Büdenbender J, Riebesell U, Form A (2011) Calcification of the Arctic coralline red algae *Lithothamnion glaciale* in response to elevated CO_2_ . Mar Ecol Prog Ser 441: 79–87.

[62] Gao K, Aruga Y, Asada K, Ishihara T, Akano T, et al (1991) Enhanced growth of the red alga *Porphyra yezoensis* Ueda in high CO_2_ concentrations. J Appl Phycol 3(4): 355–362.

[63] Zou D & Gao K (2009) Effects of elevated CO_2_ on the red seaweed *Gracilaria lemaneiformis* (Gigartinales, Rhodophyta) grown at different irradiance levels. Phycologia 48(6): 510–517.

[64] Falkenberg L, Russell B and Connell S (2012). Contrasting resource limitations of marine primary producers: implications for competitive interactions under enriched CO_2_ and nutrient regimes. Oecologia: 1–9. 10.1007/s00442-012-2507-5 23111809

[65] Miles H, Widdicomb S, Spicer JI, Hall-Spencer J (2007) Effects of anthropogenic seawater acidification on acid–base balance in the sea urchin *Psammechinus miliaris* . Mar Pollut Bull 54(1): 89–96. 17083950 10.1016/j.marpolbul.2006.09.021

[66] Kurihara H (2008) Effects of CO_2_-driven ocean acidification on the early developmental stages of invertebrates. Mar Ecol Prog Ser 373: 275–284.

[67] Levitan DR (1991) Skeletal changes in the test and jaws of the sea urchin *Diadema antillarum* in response to food limitation. Mar Biol 111: 431–435.

[68] Levitan DR, Horst CP, Fogarty ND (2007) The risk of polyspermy in three congeneric sea urchins and its implications for gametic incompatibility and reproductive isolation. Evolution 61 8: 2007–2014. 10.1111/j.1558-5646.2007.00150.x 17683441

[69] Ebert TA (1980) Relative growth of sea urchin jaws: an example of plastic resource allocation. Bull Mar Sci 30: 467–474.

[70] Widdicombe S & Spicer JI (2008) Predicting the impact of ocean acidification on benthic biodiversity: What can animal physiology tell us? JEMBE 366: 187–197.

[71] Sala E (1997) Fish predators and scavengers of the sea urchin *Paracentrotus lividus* in protected areas of the north-west Mediterranean Sea. Mar Biol 129: 531–539.

[72] Strathmann RR (1981) The role of spines in preventing structural damage to echinoid tests. Paleobiology:400–406.

[73] Bulleri F, Bertocci I, Micheli F (2002) Interplay of encrusting coralline algae and sea urchins in maintaining alternative habitats. Mar Ecol Prog Ser 243: 101–109.

[74] Coleman MA (2003) Effects of ephemeral algae on coralline recruits in intertidal and subtidal habitats. JEMBE 282(1–2): 67–84.

[75] Arenas F, Sánchez I, Hawkins S, Jenkins SR (2006) The invasibility of marine algal assemblages: role of functional diversity and identity. Ecology 87(11): 2851–2861. 17168029 10.1890/0012-9658(2006)87[2851:tiomaa]2.0.co;2

[76] Bulleri F, Tamburello L, Benedetti-Cecchi L (2009) Loss of consumers alters the effects of resident assemblages on the local spread of an introduced macroalga. Oikos 118(2): 269–279.

[77] Maggi E, Bertocci I, Vaselli S, Benedetti-Cecchi L (2011) Connell and Slatyer's models of succession in the biodiversity era. Ecology 92(7): 1399–1406. 21870613 10.1890/10-1323.1

[78] Halpern BS, Walbridge S, Selkoe KA, Kappel CV, Micheli F, et al (2008) A global map of human impact on marine ecosystems. Science 319(5865): 948–952. 18276889 10.1126/science.1149345

[79] Halpern BS, McLeod KL, Rosenberg AA, Crowder LB (2008) Managing for cumulative impacts in ecosystem-based management through ocean zoning. Ocean Coast Manag 51: 203–211.

[80] Sala E, Ballesteros E, Dendrinos P, Di Franco A, Ferretti F, et al (2012) The structure of mediterranean rocky reef ecosystems across environmental and human gradients, and conservation implications. PLoS ONE 7(2): e32742. 22393445 10.1371/journal.pone.0032742 PMC3290621

[81] Falkenberg LJ, Russell BD, Connell SD (2012) Stability of Strong Species Interactions Resist the Synergistic Effects of Local and Global Pollution in Kelp Forests. PLoS ONE 7(3): e33841 doi:10.1371/journal.pone.0033841. 22439005 10.1371/journal.pone.0033841 PMC3306304

